# Supplementary material for: Mechanisms governing the pioneering and redistribution capabilities of the non-classical pioneer PU.1
Source: Nat Commun. 2020 Jan 21;11:402. doi: 10.1038/s41467-019-13960-2 (PMC6972792; doi:10.1038/s41467-019-13960-2)
Supplement: Supplementary file 7 — Source data [file 41467_2019_13960_MOESM7_ESM.zip › Source_Data/FigureS2/FigureS2B_MotifScanOutput/KmeansCluster7/homerResults/motif22.similar.html]

motif22

## Information for motif22

A
C
G
T
T
C
G
A
T
C
G
A
T
C
A
G
A
G
C
T
A
C
G
T
A
G
C
T
A
G
T
C
A
G
T
C
C
G
A
T
C
T
A
G
C
T
G
A
T
A
C
G
  
Reverse Opposite:  

A
T
G
C
A
G
C
T
G
A
T
C
C
G
T
A
T
C
A
G
C
T
A
G
C
T
G
A
T
C
G
A
C
T
G
A
A
G
T
C
A
G
C
T
A
C
G
T
G
T
C
A
  

|  |  |
| --- | --- |
| p-value: | 1e-51 |
| log p-value: | -1.196e+02 |
| Information Content per bp: | 1.886 |
| Number of Target Sequences with motif | 102.0 |
| Percentage of Target Sequences with motif | 3.18% |
| Number of Background Sequences with motif | 147.0 |
| Percentage of Background Sequences with motif | 0.34% |
| Average Position of motif in Targets | 102.7 +/- 77.6bp |
| Average Position of motif in Background | 102.5 +/- 70.6bp |
| Strand Bias (log2 ratio + to - strand density) | 0.1 |
| Multiplicity (# of sites on avg that occur together) | 1.00 |
| Motif File: | file (matrix) reverse opposite |

### Similar de novo motifs found

|  |  |  |  |  |  |  |  |
| --- | --- | --- | --- | --- | --- | --- | --- |
| Rank | Match Score | Redundant Motif | P-value | log P-value | % of Targets | % of Background | Motif file |
| 1 | 0.959 | C A G T T G C A T C G A T C A G A C G T A C G T A G C T A G T C A G T C C G A T C T A G C T G A | 1e-47 | -109.245255 | 3.24% | 0.41% | motif file (matrix) |
| 2 | 0.834 | A T C G A C G T C G T A T C G A T C A G A C G T A C G T A C G T A G T C G A T C G C A T | 1e-41 | -94.622054 | 3.18% | 0.49% | motif file (matrix) |
| 3 | 0.822 | A C G T C G T A C T G A A C T G A C G T A C G T A C G T A G T C A T G C | 1e-26 | -60.142132 | 2.55% | 0.53% | motif file (matrix) |
